# Co‐expression of human calreticulin significantly improves the production of HIV gp140 and other viral glycoproteins in plants

**DOI:** 10.1111/pbi.13369

**Published:** 2020-03-13

**Authors:** Emmanuel Margolin, Youngjun J. Oh, Matthew Verbeek, Jason Naude, Daniel Ponndorf, Yulia Alexandrovna Meshcheriakova, Hadrien Peyret, Michiel T. van Diepen, Ros Chapman, Ann E. Meyers, George Peter Lomonossoff, Nobuyuki Matoba, Anna‐Lise Williamson, Edward P. Rybicki

**Affiliations:** ^1^ Division of Medical Virology Department of Pathology Faculty of Health Sciences University of Cape Town Cape Town South Africa; ^2^ Wellcome Trust Centre for Infectious Disease Research in Africa University of Cape Town Cape Town South Africa; ^3^ Faculty of Health Sciences Institute of Infectious Disease and Molecular Medicine University of Cape Town Cape Town South Africa; ^4^ Biopharming Research Unit Department of Molecular and Cell Biology University of Cape Town Cape Town South Africa; ^5^ Department of Pharmacology and Toxicology University of Louisville School of Medicine Louisville KY USA; ^6^ Department of Biological Chemistry John Innes Centre Norwich UK

**Keywords:** glycoprotein, chaperone, folding, cleavage, calreticulin, furin, co‐expression, HIV, virus

## Abstract

Plant molecular farming (PMF) is rapidly gaining traction as a viable alternative to the currently accepted paradigm of producing biologics. While the platform is potentially cheaper and more scalable than conventional manufacturing systems, expression yields and appropriate post‐translational modifications along the plant secretory pathway remain a challenge for certain proteins. Viral fusion glycoproteins in particular are often expressed at low yields in plants and, in some cases, may not be appropriately processed. Recently, however, transiently or stably engineering the host plant has shown promise as a strategy for producing heterologous proteins with more complex maturation requirements. In this study we investigated the co‐expression of a suite of human chaperones to improve the production of a human immunodeficiency virus (HIV) type 1 soluble gp140 vaccine candidate in *Nicotiana benthamiana* plants. The co‐expression of calreticulin (CRT) resulted in a dramatic increase in Env expression and ameliorated the endoplasmic reticulum (ER) stress response ‐ as evidenced by lower transcript abundance of representative stress‐responsive genes. The co‐expression of CRT similarly improved accumulation of glycoproteins from Epstein‐Barr virus (EBV), Rift Valley fever virus (RVFV) and chikungunya virus (CHIKV), suggesting that the endogenous chaperone machinery may impose a bottleneck for their production. We subsequently successfully combined the co‐expression of human CRT with the transient expression of human furin, to enable the production of an appropriately cleaved HIV gp140 antigen. These transient plant host engineering strategies are a promising approach for the production of high yields of appropriately processed and cleaved viral glycoproteins.

## Introduction

Plant‐based manufacturing of recombinant proteins is an attractive platform for the production of biopharmaceuticals, with its unique advantages of scalability and cost (D'Aoust *et al.*, 2010; Tschofen *et al.*, 2016). This is mainly due to the cost‐effective growth of large amounts of plant biomass in a short time frame, but also a result of the lower infrastructure requirements for protein production in plants (Nandi *et al.*, 2016; Paul *et al.*, 2013; Rybicki, 2010). These advantages render the technology particularly appealing to developing countries where the capacity to manufacture biopharmaceuticals is often not available, and more generally, where rapid responses to pandemic outbreaks of infectious diseases may be necessary (Rybicki, 2009; Rybicki, 2010).

However, despite these unique advantages, the clinical deployment of plant‐made pharmaceuticals (PMP) has been confined to niche areas where the current paradigm of biopharmaceutical production is lacking (Paul *et al.*, 2013; Stoger *et al.*, 2014). This is partly a reflection of the low yields of many proteins during the infancy of the technology, and the realization that certain post‐translational modifications may not occur optimally in the system (Castilho et al., 2018; Gomord and Faye, 2004; Loos *et al.*, 2015; Mamedov *et al.*, 2019; Rybicki, 2010; Strasser, 2016; Streatfield, 2007; Wilbers *et al.*, 2016). The development of improved expression technologies now enables the production of most heterologous proteins in plants at reasonably high levels, although many viral glycoproteins still appear to be produced inefficiently in the system (Lomonossoff and D'Aoust, 2016; Margolin *et al.*, 2018; Peyret and Lomonossoff, 2015). This severely limits the utility of the platform for the production of vaccines against many emerging viruses and pandemic viruses which generally are thought to require the induction of antibodies against the surface viral glycoproteins to protect against infection (Murin *et al.*, 2019; Rey and Lok, 2018). We previously hypothesized that the low expression levels observed for many viral glycoproteins in plants was a reflection of the inherent differences in the endogenous chaperone machinery compared to those in the natural mammalian hosts where the viral glycoproteins are usually produced (Braakman and van Anken, 2000; Margolin *et al.*, 2018). In addition to chaperone‐mediated folding, glycosylation and proteolytic processing also serve as critical events in the maturation and folding of viral glycoproteins (Braakman and van Anken, 2000).

Following translation, nascent glycoproteins translocate into the ER where an oligosaccharide precursor is transferred to the asparagine residue of the N‐X‐S/T sequon (where X is any amino acid other than proline) (Chavan and Lennarz, 2006; Zielinska *et al.*, 2010). This is mediated by the host oligosaccharyltransferase (OST) complex, a multimeric complex that is adjacent to the protein translocon channel (Kelleher and Gilmore, 2006). The sequential removal of the outermost 2 glucose residues from the glycan by α‐glucosidase I and α‐glucosidase II yields a monoglucosylated structure which is targeted into the calnexin/calreticulin (CNX/CRT) pathway for chaperone‐mediated folding (Deprez *et al.*, 2005; Hammond *et al.*, 1994; Hebert *et al.*, 1995). CRT and its membrane‐bound homologue CNX co‐ordinate interaction of the target glycoprotein with other mediators of protein folding (Frickel *et al.*, 2002; Jessop *et al.*, 2009; Kozlov *et al.*, 2010; Wada *et al.*, 1991; Zhang and Herscovitz, 2003). Correctly folded proteins are released from the pathway following the removal of the innermost glucose by α‐glucosidase II, to continue along the secretory pathway (Hebert *et al.*, 1995). Conversely, aberrantly folded glycoproteins are reglucosylated by UDP‐glucose:glycoprotein glucosyltransferase 1, resulting in their retention for another cycle of chaperone‐mediated folding (Tannous *et al.*, 2015). Terminally misfolded proteins are targeted for ER‐associated degradation (ERAD), which involves their retrotranslocation back into the cytosol for proteosomal degradation (Williams, 2006).

Correctly folded glycoproteins are then subjected to further host‐specific glycan modifications, and sometimes proteolytic processing, along the Golgi network (Chung *et al.*, 2017; Zhou *et al.*, 1999). Proteolytic maturation of viral glycoproteins in animals and humans is most commonly mediated by furin proteases, which do not naturally occur in plants (Faye *et al.*, 2005; Pasquato *et al.*, 2013). This necessitates ectopic expression of the protease in plants to achieve appropriate cleavage of target proteins (Mamedov *et al.*, 2019; Wilbers *et al.*, 2016). Given the critical role of furin cleavage in the folding of many glycoproteins, the lack of the enzyme in plants probably contributes to the inefficient production of many viral glycoproteins (Pasquato *et al.*, 2013).

We previously reported the transient expression of soluble HIV gp140 trimers in *N. benthamiana* plants as a potentially cheaper alternative to conventional cell culture‐based production platforms (Margolin, 2018). While these antigens were immunogenic, the expression levels were poor, negating the potential cost benefit of plant‐based protein production (Rybicki, 2010). Furthermore, transient expression of the antigens caused considerable pathology in the leaves: this is consistent with the phenotype described for ER‐stress, resulting from the accumulation of misfolded proteins in plants (Hamorsky *et al.*, 2015). Similar observations have been reported for other viral glycoproteins that accumulate poorly in plants (Margolin *et al.*, 2018; Pera *et al.*, 2015; Phoolcharoen *et al.*, 2011). These observations suggest that the endogenous folding machinery may represent a bottleneck for the efficient production of many heterologous viral glycoproteins in plants.

The host folding machinery has been acknowledged as a bottleneck in prokaryotic expression platforms, and the co‐expression of chaperone proteins has been reported to improve the production of several target proteins (Georgiou and Valax, 1996; Robinson *et al.*, 1994). Surprisingly, few studies have explored the utility of this approach in plants. A single report describing the expression of *Escherichia coli* type 3 secretion chaperone CesT to support the production of the bacterial receptor protein Tir in *N. benthamiana* appears to be the only published study to investigate this approach in plants (MacDonald *et al.*, 2017). Preceding this, a patent application describing the co‐expression of the *Arabidopsis thaliana* lectin binding chaperones (CNX and CRT) reported improved expression of viral glycoproteins from Ebola, hepatitis C and influenza viruses ‐ although the improvement was modest [US 2014/0127749 A1].

We have hypothesized that divergence during their evolution of plants from the natural mammalian hosts of many viruses may have resulted in incompatibility between the target glycoprotein and the plant homologues of critical chaperones required to mediate protein folding (Margolin *et al.*, 2018). In this study, we explored the co‐expression of human chaperone proteins as an approach to improve the production of a candidate HIV gp140 antigen. We subsequently demonstrated the broad applicability of this approach by improving the yield of several other viral glycoproteins, many of which could not in fact be detectably produced in the absence of chaperone co‐expression. Finally, we combined the co‐expression of CRT with the expression of human furin to enable the production of an appropriately cleaved HIV‐1 gp140 antigen.

## Results

### Putative plant homologues of human ER‐resident chaperones have low sequence identity compared to the human proteins

In order to develop a basis for the hypothesis that poor HIV Envelope (Env) accumulation in plants was due to incompatibility of the viral glycoprotein with the endogenous chaperone machinery, we interrogated the *N. benthamiana* genome for homologues of key human molecular chaperones that are known to be involved in protein folding. These included CNX and CRT – which are known to associate with HIV Env during folding – as well as protein disulfide‐isomerase (PDI), ER resident protein 57 (ERp57) and binding‐immunoglobulin protein (BiP) (Otteken and Moss, 1996). Considerable amino acid sequence divergence ‐ ranging from 34‐70% ‐ was observed for all the chaperones of interest, highlighting that fundamental differences exist between the chaperone folding machinery of human cells compared to *N. benthamiana* (Table 1). Plant homologues of both CRT and CNX, the co‐ordinators of chaperone‐mediated glycoprotein folding, had low levels of sequence identity compared to the human versions. This was even more pronounced for the oxidoreductases PDI and ERp57, which are required for the formation of disulphide bridges. The sequence of BiP was more conserved, with the plant protein retaining 71% identity to the human protein. These observations suggest that the endogenous plant chaperone machinery may not support the efficient production of the HIV Env glycoprotein, which is dependent on the CNX/CRT folding cycle and contains extensive disulphide bonds (Land *et al.*, 2003; Otteken and Moss, 1996). These observations may also explain the inefficient production of other viral glycoproteins which may have similar folding requirements.

**Table 1:** Sequence identity of *N. benthamiana* homologues of key human molecular chaperones. The hit with the greatest sequence identity is reflected for each human protein

Chaperone	UniProt accession no.	Identity (%)	E value
Calnexin	P27824	42.86	e^‐123^
Calreticulin	P27797	55.68	e^‐124^
BiP	P11021	70.85	0.0
ERp57	P30101	34.5	9e^‐78^
PDI	P07237	38.68	1e^‐96^

### Co‐expression of human CRT improves the production of a soluble HIV‐1 gp140 antigen

**Figure 1:**
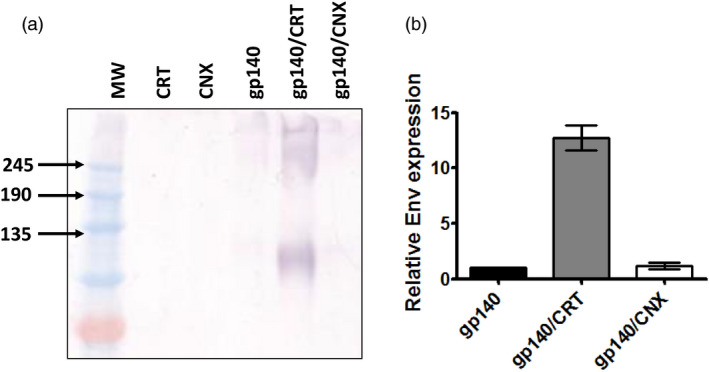
Co‐expression of the human molecular chaperone calreticulin with HIV‐1 Env gp140 improves accumulation in plants. (a) Western blot analysis of crude leaf protein homogenate, following the co‐expression of HIV Env gp140 with CRT and CNX. Equal amounts of total soluble protein were resolved by SDS‐PAGE and immunoblotted using polyclonal goat anti‐gp120 antibody to detect the recombinant HIV antigen. (b) Relative expression of HIV Env gp140 protein following the co‐expression of CRT and CNX. Recombinant protein expression in the presence of co‐expressed CRT and CNX is reflected relative to expression of the protein in the absence of any co‐expressed chaperone. Relative expression levels were determined by gel densitometry following western blotting and were determined from 3 separate infiltrations. (MW = molecular weight marker, CRT = CRT only, CNX = CNX only, gp140/CRT = co‐expression of gp140 and CRT, gp140/CNX = co‐expression of gp140 and CNX).

The low levels of sequence identity observed for the plant versions of the human chaperones prompted us to co‐express the human proteins to support the production of HIV gp140. Recombinant *A. tumefaciens* strains encoding each of the chaperones were vacuum infiltrated with the strain expressing the HIV gp140 antigen at a 1:1 ratio. Expression of PDI on its own resulted in severe tissue necrosis by 3 days post infiltration (Figure S1), even at low culture densities. This precluded efficient extraction of protein from agroinfiltrated plant leaves and was not pursued any further. Co‐expression of CRT resulted in a dramatic improvement in Env gp140 protein accumulation (Figure 1a), and a slight improvement was also observed for CNX (Figure 1b). Although the levels of expression were increased substantially using CRT, there was no change in the levels of unresolved higher molecular weight aggregates which also appeared to increase proportionately. Increasing the bacterial inoculum encoding CRT failed to impact the formation of these aggregates. Gel densitometry of serially diluted crude protein samples yielded a 12.7‐fold increase in relative expression of the desired gp140 band in the presence of co‐expressed CRT, and 1.17‐fold in the presence of co‐expressed CNX (Figure 1b) based on 3 independent infiltrations. It is interesting to note that the signal observed by western blotting (Figure 1a) was slightly smaller than expected for gp140. This may be due to a lower glycan occupancy in plants, although this needs to be determined experimentally (Castilho et al., 2018). Neither BiP nor ERp57 co‐expression resulted in any discernible improvement in Env expression, even when co‐expressed with CRT (Figure S2).

### Co‐expression of human CRT ameliorates the ER‐stress response following HIV gp140 expression

**Figure 2:**
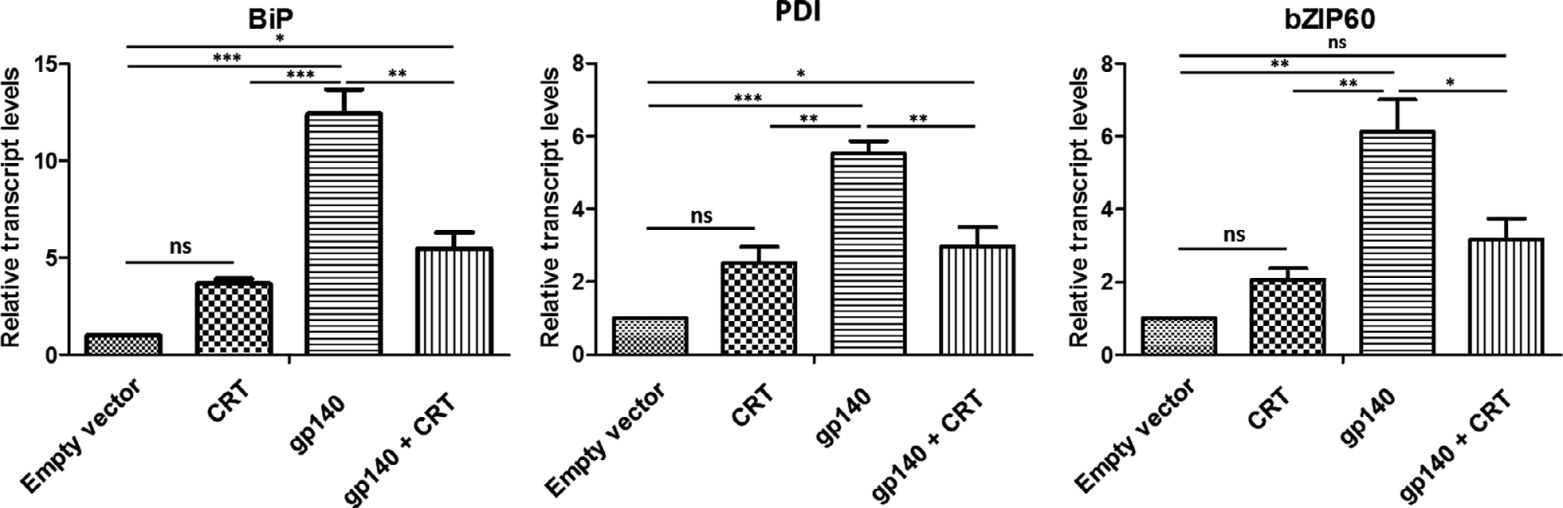
qRT‐PCR analysis of ER stress related genes. The mean fold‐increase of (a) *BiP*, (b) *PDI* and (c) *bZIP60* are indicated following the co‐expression of human calreticulin with HIV gp140. The 18S rRNA was used for the normalization of cDNA amount. A control, comprising of plants infiltrated with *A. tumefaciens*, transformed with the empty pEAQ‐*HT* vector*,* was included for comparison. Error bars represent standard deviation of three biological replicates. (*; *P* < 0.05, **;* P* < 0.01, ***;* P* < 0.001, NS = not significant). (empty vector = infiltration with *A. tumefaciens* transformed with pEAQ‐*HT*, CRT = CRT expression only, gp140 = gp140 expression only, CRT + gp140 = co‐expression of gp140 and CRT).

Given the divergence of the plant and human chaperone machinery it is plausible that the HIV Env glycoprotein is inefficiently folded in plants, leading to ER‐stress. This would account for the pathology that we previously reported following transient expression of Env gp140 (Margolin *et al.*, 2019). Considering the dramatic improvement in Env production following CRT co‐expression, we were interested in determining how this impacted the ER stress response. We therefore performed RT‐qPCR to determine the relative transcript abundance of *BiP*, *PDI* and *bZIP60,* which have been reported to serve as representative markers of the plant ER stress response (Hamorsky *et al.*, 2015). As well as improving the relative levels of Env production *in planta* by almost 13‐fold, co‐expression of CRT with gp140 resulted in a significant decrease in expression of *BiP*, *PDI* and *bZIP60,* by 2.26, 1.85, and 1.93 fold respectively, compared to plants where the glycoprotein was expressed without the chaperone (Figure 2). These data suggest that in the presence of the co‐expressed chaperone, folding of Env was improved and the ER stress response of the host plant was reduced. Interestingly, expression of the chaperone alone exhibited a trend towards increased expression of all 3 ER‐stress genes, although this was not found to be statistically significant.

### Co‐expression of human CRT improves the accumulation of diverse viral glycoproteins

**Figure 3:**
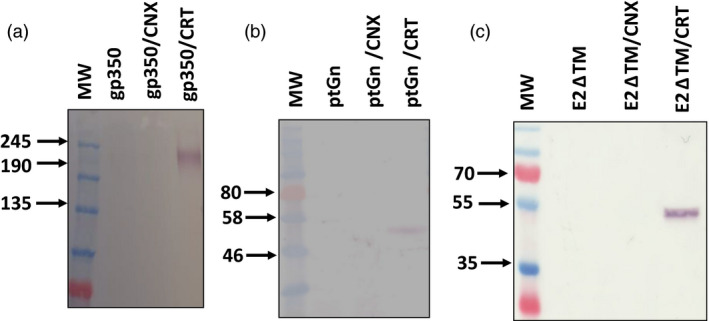
Broad applicability of chaperone co‐expression with diverse viral glycoproteins. Western blotting was performed to detect expression of EBV gp350_Ecto_ (a), RVFV ptGn (b), ChikV E2ΔTM (c) following co‐expression of human chaperones. Equal quantities of total soluble protein were resolved by SDS‐PAGE to allow for comparison. Recombinant EBV gp350 and CHIKV E2∆TM was detected with monoclonal mouse anti‐histidine antibody whereas RVFV ptGn was detected using polyclonal rabbit antibodies raised against a synthetic peptide in the glycoprotein. (EBV gp350 Epstein‐Barr virus gp350 ectodomain, ptGn = Rift Valley fever virus Gn glycoprotein, E2ΔTM = soluble chikungunya virus E2 glycoprotein, MW = molecular weight marker, gp350 = gp350 expression only, gp350/CNX = co‐expression of gp350 and CRT, gp350/CRT = co‐expression of gp350 and CRT, ptGn = Gn only, ptGn/CNX = co‐expression of ptGn and CNX, ptGn/CRT = co‐expression of ptGn and CRT, E2ΔTM = expression of E2ΔTM only, E2ΔTM/CNX = co‐expression of E2ΔTM and CNX, E2ΔTM/CRT = co‐expression of E2ΔTM and CRT).

Following the observation that CRT improved the accumulation of HIV gp140, we investigated this approach for expression of other viral glycoproteins which may have a similar reliance on the host chaperone machinery for folding. We also co‐expressed human CNX with each glycoprotein as the membrane‐bound homologue may preferentially mediate folding of membrane‐associated glycoproteins (Wada *et al.*, 1991). Specifically, we tested this approach for the glycoprotein antigens Epstein‐Barr virus (EBV) gp350, chikungunya virus (CHIKV) E2, Rift Valley fever virus (RVFV) Gn, dengue virus (DenV) E and influenza H1 HA. The EBV gp350 and CHIKV E2 were both truncated to remove their transmembrane and cytoplasmic domains (subsequently referred to as EBV gp350_Ecto_ and CHIKV E2ΔTM, respectively). The H1 HA protein was the full length antigen, expression of which has been reported elsewhere (Margolin, 2018). The DenV E protein contained the full length glycoprotein precursor as encoded by the pre‐membrane and envelope coding sequence (prME). Co‐expression of CRT improved the accumulation of all the antigens tested (Figure 3), with the exception of influenza H1 HA and DenV E proteins (Figure S3). In the case of the EBV gp350_Ecto_, RVFV Gn and CHIKV antigens, expression of the glycoproteins could only be detected following co‐expression of the chaperone under the conditions that were tested (Figure 3a‐c). Co‐expression of CNX did not appear to have a discernible impact on the accumulation of any of the antigens tested.

### Combining expression of CRT and human furin enables the production of a cleaved HIV‐1 gp140 SOSIP.664 antigen

**Figure 4:**
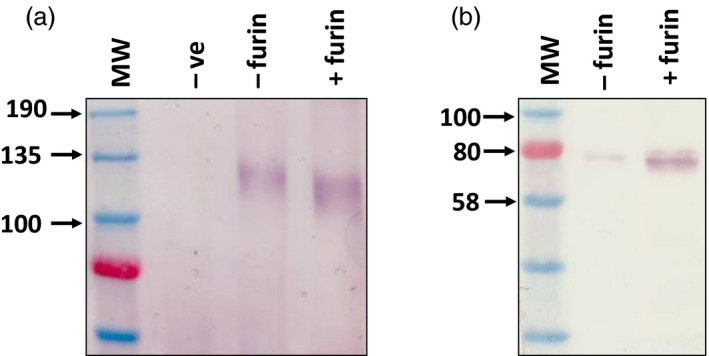
Production of processed CAP256 SU gp140 SOSIP.664 by co‐expression of human furin *in planta*. Western blot to detect expression of HIV gp140 (a) and influenza HA (b), following co‐expression of human furin. Recombinant HIV and influenza glycoproteins were detected using polyclonal goat and rabbit antibodies respectively. Human CRT was co‐expressed with the HIV Env glycoprotein in both experimental samples (‐ furin/+furin) to enable the production of the antigen at detectable levels. The influenza HA antigen was not co‐expressed with any chaperone. (In a: ‐ve = infiltration with *A. tumefaciens* transformed with pEAQ‐*HT*, ‐furin = gp140 and CRT co‐expression, +furin = gp140, CRT and furin co‐expression and in b: ‐furin = HA expression only, +furin = HA and furin co‐expression).

The lack of naturally occurring furin in plants complicates the production of authentic viral glycoproteins which may not be properly processed without the protease (Faye *et al.*, 2005; Pasquato *et al.*, 2013). In our previous study we circumvented the need for furin cleavage of HIV Env by replacing the cleavage site with a glycine‐rich flexible linker peptide (Margolin *et al.*, 2019). In addition to improving the expression of this cleavage‐independent Env antigen by co‐expressing chaperones, we were also interested in exploring the production of a fully cleaved HIV gp140. Cleavage of the glycoprotein plays an important role in the folding of the protein and the prototypic BG505 SOSIP.664 used in clinical testing was produced by co‐expression of human furin to promote optimal processing (Ringe *et al.*, 2013; Sanders *et al.*, 2013). Although two previous studies have reported the expression of furin in plants to facilitate the processing of target proteins, this has not been reported for a viral glycoprotein (Mamedov *et al.*, 2019; Wilbers *et al.*, 2016). We therefore designed an HIV Env antigen based on the cleaved “SOSIP” antigen design described for the prototypic Env vaccine BG505 SOSIP.664, which is being evaluated in clinical trials (Sanders *et al.*, 2013; Sanders et al., 2015). Two changes were made to the HIV gp140 antigen described previously (Margolin *et al.*, 2019) to generate the SOSIP protein: a multi‐basic (RRRRRR) cleavage site was included in place of the flexible linker and an artificial disulphide bridge was added to stabilize the association of gp41 and gp120 following cleavage (Binley *et al.*, 2000; Binley *et al.*, 2002; Sanders *et al.*, 2002). Transient expression of the antigen and CRT with human furin resulted in a 15–20 kDa size shift corresponding to the cleavage of a 157 amino acid fragment (Figure 4a). In addition, the protease was also co‐expressed with the influenza H1 HA and DenV prME antigens to determine if the correctly processed glycoproteins could be produced in plants. Unexpectedly, following furin co‐expression with these proteins there was no evidence of cleavage of the glycoprotein which is expected to yield products of approximately 48 kDa and 28 kDa respectively (Figure 4b).

## Discussion

It has been argued that plants are a promising expression host for the production of recombinant proteins, given the ability to reproduce mammalian‐type post‐translational modifications. However, it is also apparent that certain post‐translational modifications of proteins may not occur optimally along the plant secretory pathway (Faye *et al.*, 2005). This may account for the low expression yields that have been observed for several viral glycoproteins which require carefully coordinated folding by ER‐resident chaperones, proteolytic processing and ‐ in some cases ‐ extensive glycosylation (Braakman and van Anken, 2000; Hebert *et al.*, 1995; Pasquato *et al.*, 2013). Given the divergent evolution of plants from the natural mammalian hosts of many viruses, fundamental differences in the plant folding machinery may result in inefficient production of certain proteins. We have reported that a prototype HIV gp140 antigen which accumulated at low levels in plants, was prone to aggregation and induced pathology that is consistent with ER‐stress (Hamorsky *et al.*, 2015). Based on these observations, we speculated that the chaperone machinery *in planta* may be different to the mammalian chaperones that are required to mediate folding of the protein. Alternately, the endogenous levels of key chaperones in plants may be too low to efficiently mediate the folding of certain viral glycoproteins.

In this study we showed that putative plant homologues of key human ER‐resident chaperones that are known to mediate glycoprotein folding have low levels of identity with the human versions. Therefore, it is conceivable that the endogenous plant folding machinery may not efficiently mediate folding of HIV Env and other similarly complex animal‐infecting viral glycoproteins. In order to address this, we co‐expressed a suite of human chaperones with HIV gp140, in the hope of improving the production of the protein by providing the appropriate folding machinery. These chaperones were all successfully expressed in plants except for PDI which resulted in severe tissue necrosis. Although the mechanism for this is not clear, the optimized coding sequence may have been poorly recognized by the plant translational machinery resulting in misfolding of the protein. An alternative explanation could be that the high expression of the mammalian PDI driven by pEAQ‐HT resulted in toxicity; the nature of this multifunctional enzyme family between plant and mammalian‐derived PDS is very diverse (Selles *et al.*, 2011). Human CRT dramatically improved the accumulation of the glycoprotein, and similarly improved production of several other viral glycoproteins which are under development as vaccine immunogens or reagents. This enabled the purification of up to 21.5 mg purified Env trimer/kg of plant biomass which previously accumulated below 2 mg/kg (Margolin *et al.*, 2019). The CHIKV and RVFV glycoproteins described in this study could not in fact be expressed at detectable levels in the absence of the co‐expressed chaperone. These data suggest that the endogenous plant chaperone machinery may impose a critical bottleneck for the production of certain mammalian viral glycoproteins. In addition to improving the accumulation of HIV gp140, co‐expression of CRT also reduced the expression of representative markers of ER stress. This observation is quite remarkable, as it implies that despite approximately 13‐fold improvement in expression of the protein, the levels of misfolded protein were reduced following co‐expression of the chaperone. Interestingly, this approach did not improve the production of influenza HA but rather appeared to reduce expression. It is possible that high levels of co‐expressed human CNX and CRT may have competed with plant chaperones for the binding to HA, therefore impeding the folding process. It is also unclear where these proteins were located along the secretory pathway as western blotting was performed on crude leaf homogenate. Whilst each glycoprotein contained a signal peptide to direct the antigen into the secretory pathway it is also plausible that misfolded protein could have been retained in the CNX/CRT folding pathway. Further work is needed to confirm the localization of the recombinant viral glycoproteins.

The transient co‐expression of both CRT and furin enabled the production of an appropriately cleaved “SOSIP”‐type HIV gp140 antigen in plants. Although two previous studies have reported the co‐expression of the protease in plants for the targeted processing of heterologous proteins, this is the first report to our knowledge of furin‐mediated maturation of a viral glycoprotein in a plant system (Mamedov *et al.*, 2019; Wilbers *et al.*, 2016). Thus, this study establishes an important precedent for the expression of other proteases which may be lacking along the secretory pathway in plants. The co‐expression of furin did not result in the cleavage of the H1 HA glycoprotein. Prediction software did not recognize the cleavage site efficiently *in silico*. This may therefore be a suboptimal cleavage site which is not well recognized by the protease *in vivo*. Further experiments are planned to produce furin‐cleaved glycoproteins from other viruses, and to compare the impact of the enhanced cleavage motif used in this study with the native furin recognition sequence. This approach has been reported to improve cleavage of a mammalian cell‐produced HIV gp140 and may be a useful way to improve furin‐processing in plants following co‐expression of the protease (Binley *et al.*, 2002).

Animal experiments will be necessary to assess the immunogenicity of the antigens produced in this study. It will be interesting to assess if the HIV gp140 antigens that were produced in the presence of co‐expressed CRT will elicit better neutralizing antibodies compared to the protein that was previously produced in the absence of the chaperone (Margolin *et al.*, 2019). The RVFV Gn and CHIKV E2 antigens are both promising vaccine candidates, as recombinant versions of these proteins have been reported to protect against challenge in mice (de Boer *et al.*, 2010; Kumar *et al.*, 2012). While a monomeric EBV gp350 may not be an ideal vaccine candidate, it is encouraging that such a large and heavily glycosylated protein could be successfully expressed in the system (Cui *et al.*, 2016; Kanekiyo *et al.*, 2015; Moutschen *et al.*, 2007). The full length glycoprotein contains 907 amino acids and has 37 putative N‐glycosylation sites. These soluble glycoproteins are also excellent candidates for multimerization on synthetic nanoparticles to improve their immunogenicity (Smith *et al.*, 2015). This approach has already shown promise for improving the vaccine‐elicited immune responses against EBV gp350, which is poorly immunogenic (Kanekiyo *et al.*, 2015).

To our knowledge, this is the first study to report the expression of a human chaperone protein in a plant expression system, and the first to show improved production of a viral glycoprotein with this approach. Similarly, this is the first report to demonstrate cleavage of a viral glycoprotein *in planta* by co‐expressing a heterologous protease. Further experiments are planned to expand the range of chaperones that are co‐expressed with target proteins, and to explore this approach for additional glycoproteins. In this study we have specifically focused on ER‐resident chaperones due to their central role in coordinating the folding of viral glycoproteins. However, the co‐expression of chaperones that act at other stages along the secretory pathway may also have similar value. This may even be a useful way to improve the production of virus‐like particles in cases where assembly may require a chaperone‐mediated process (Chromy *et al.*, 2003). The results presented in this study highlight the value of remodelling the plant secretory pathway for the production of recombinant proteins and serve as a basis for future engineering strategies that will be implemented to improve the production of viral glycoprotein‐based vaccines in plants.

## Methods

### Bioinformatics analysis of putative *N. benthamiana* homologues of human chaperones

The sequence identities of homologous plant chaperones were determined by interrogating the *Nicotiana benthamiana* genome (V1.0.1) with the amino acid sequences of human chaperones (Fernandez‐Pozo *et al.*, 2015). In each case the sequence was blasted against predicted proteins (blastp) from *N. benthamiana* and the hit with the greatest sequence identity was reflected.

### Gene design and assembly of expression cassettes

A soluble variant of the EBV gp350 glycoprotein (UniProt accession #P03200) was conceived by removing the transmembrane and cytoplasmic domains of the antigen to generate gp350_Ecto_. The gp350_Ecto_ coding sequence was synthesized in frame with the murine monoclonal antibody‐derived LPH leader, the furin cleavage site was replaced with 2 repeats of a glycine‐rich flexible linker (GGGGS) and a 6 × His tag was added to the C terminus of the protein. A soluble consensus CHIKV E2 glycoprotein sequence, lacking the transmembrane region (∆TM), was generated from the extracellular portion of the E2 protein from 3 isolates (GenBank accession: HM045792.1, HM045795.1, HM045805.1). The CHIKV E2∆TM antigen was synthesized without the native leader sequence, to enable the assembly cloning of the gene with a heterologous signal peptide, and with a 6 × His tag at the C terminus following a linker peptide (EAAAKA). The HIV gp140 SOSIP.664 Env antigen was designed from the CAP256 superinfecting virus that was used previously (Margolin *et al.*, 2019). The coding sequence was modified to contain a hexa‐arginine (RRRRRR) motif in place of the native cleavage site and other stabilizing mutations that have been reported elsewhere (Sanders *et al.*, 2013). The DenV PrME coding sequence was PCR amplified from a synthetic version of the full length structural protein open reading frame (strain Hawaii, GenBank: KM204119.1) (FWD: 5’CCTTACCGGTAACAATGCTTCTTATGCTGCTGCCTAC and RVS: 5’ GAAACTCGAGCTAAGCCTGCACCATAACACCCAG). The template used for PCR was codon optimized for expression in *N. benthamiana*. The coding sequences for human CRT (UniProt accession #P27797), CNX (UniProt accession #P27824), BiP (UniProt accession #P11021), ERP57 (UniProt accession #P30101) and PDI (UniProt accession #P07237) were synthesized by GenScript with their native signal peptides to enable their natural targeting to the secretory pathway as required for their role in protein folding. All chaperone sequences were optimized to reflect the preferred human codon bias. The CHIKV antigen was synthesized to reflect the plant codon preference whereas the remaining genes were synthesized to reflect the optimal human codon usage. Synthetic *AgeI* and *XhoI* sites were introduced at the 5’ and 3’ ends of all gene sequences except CHIKV E2∆TM which was flanked by artificial *Nco*I and *Xho*I restriction sites. The CHIKV E2∆TM antigen was cloned into pTRAkc‐ERH, in frame with the LPH leader sequence that is present in the plasmid, using Nco1 and Xho1. All other genes were cloned into pEAQ‐*HT* (GenBank accession no: GQ497234.1) using *Age*I and *Xho*I (Sainsbury *et al.*, 2009). The full length human furin sequence was PCR amplified from pcDNA3.1, without further modification (FWD: 5’ CTACAACCGGTATGGAGCTGAGGCCCTGG 3’and RVS: 5’ AAGTGCTCGAGTCAGAGGGCGCTCTGGTC 3’) and cloned into pEAQ‐*HT* using artificial *Age*I and *Xho*I sites that were included in the primers (Binley *et al.*, 2002). The recombinant pEAQ‐HT and pTRAkc‐ERH (Maclean *et al.*, 2007) plasmids were electroporated into *A. tumefaciens* AGL1 and GV3101::pMP90RK respectively. Putative transformants were verified by PCR using vector‐specific primers as previously reported (Margolin *et al.*, 2019). Recombinant *A. tumefaciens* strains encoding RVFV ptGn and HIV‐1 CAP256 SU gp140 have been described elsewhere (Margolin *et al.*, 2019; Mbewana, 2017).

### Transient co‐expression of viral glycoproteins with chaperones and furin *in planta*

Glycerol stocks of recombinant *A. tumefaciens* were grown in 10 ml Luria broth (LB) and scaled up to 1 litre in LB base medium as previously described (Margolin *et al.*, 2019). *A. tumefaciens* strains transformed with pEAQ‐*HT* expression plasmids were selected for using 50 µg/mL kanamycin (Sigma‐Aldrich St Louis, Missouri) and 50 µg/mL carbenicillin (Sigma‐Aldrich) whereas *A. tumefaciens* GV3101::pMP90RK (pTRAkc‐ERH: CHIKV E2∆TM) was selected for using 50 µg/mL rifampicin, 50 µg/mL carbenicillin and 30 µg/mL kanamycin (Sigma‐Aldrich). Growth media was supplemented with 20 µm acetosyringone in the final culture step. Rifampicin was omitted from the final culture step for *A. tumefaciens* GV3101::pMP90RK (pTRAkc‐ERH: CHIKV E2∆TM). The bacterial inoculum was adjusted to a final OD_600_ of 0.5 for each construct using resuspension medium (10 mm MgCl_2_, 10 mm MES [pH5.6], 200 µm acetosyringone), except for RVFV ptGn which was previously reported to accumulate optimally at OD_600_ = 0.25. Whole *N. benthamiana* plants were vacuum infiltrated with the bacterial suspension as reported previously (Margolin *et al.*, 2019).

### Small scale extraction of crude leaf lysate

Crude leaf protein was harvested from groups of 3 plants to account for biological variability. Clippings were taken from 3 leaves on each plant, 5 days post infiltration, and combined. The leaf material was crushed in liquid nitrogen and then resuspended in 3 buffer volumes of phosphate buffered saline [pH 7.4], supplemented with cOmplete™ EDTA‐free protease inhibitor. The lysate was incubated at 4°C, for 1 h, with shaking and then clarified as described previously (Margolin *et al.*, 2019). The supernatant was quantified using the *DC* protein assay (Bio‐Rad, Irvine, CA).

### Quantification of relative glycoprotein expression levels

Equal amounts of total soluble protein were resolved by SDS‐PAGE and then immunoblotted as previously described. The recombinant chaperones were detected using 1:5000 dilutions of rabbit polyclonal anti‐CRT (Abcam, ab2907, Cambridge, UK), rabbit polyclonal anti‐CNX (Abcam, ab75081), mouse monoclonal anti‐PDI (Abcam, ab2792), rabbit monoclonal anti‐GRP78 (Abcam, ab108615) or rabbit polyclonal to ERp57 (Abcam, ab10287). HIV gp140 was detected as previously described (Margolin *et al.*, 2019). Recombinant RVFV ptGn, and DenV E proteins were detected using polyclonal rabbit antibodies that were raised against synthetic peptide sequences from the glycoproteins (RVFV: CFEHKGQYKGTMDSGQTKRE and DenV: CTGDQHQVGNETTEH, GenScript) (de Boer *et al.*, 2012). HA was detected using rabbit polyclonal anti‐HA1 H1N1 A/California/14/2009 (Abcam, ab90602). CHIKV E2∆TM and EBV gp350_Ecto_ was detected using 1:2000 dilution of monoclonal mouse anti‐histidine (Serotech, MCA1396). Furin was detected using 1:5000 polyclonal rabbit anti‐furin antibody (Abcam, AB3467). In turn, the primary antibodies were detected with 1:10 000 anti‐rabbit IgG‐alkaline phosphatase (Sigma, A3687) and 1:10 000 goat anti‐mouse IgG‐alkaline phosphatase (Sigma, A3562). The relative glycoprotein expression levels were determined by gel densitometry following western blotting. Images were captured using the BioRad Molecular Imager® Gel Doc™ XR + System and analysed using Image Lab™ Software (V5.2.1). Individual lanes were defined manually and the software protocol run for low intensity bands. Saturated bands were excluded for quantification. The relative expression levels were adjusted for the dilution factor where necessary. Results were presented as the mean of 3 independent infiltration experiments.

### RT‐qPCR analysis of ER stress‐related genes

Total RNA was extracted from 100 mg of leaf tissue 48 hours after agroinfiltration. Leaf material was homogenized in liquid nitrogen, using a mortar and pestle, followed by RNA extraction using the QIAShredder (Qiagen, Hilden, Germany) and RNAqueous Phenol‐free total RNA Isolation Kits (Thermo Fisher Scientific, Waltham, MA). Following RNA extraction, residual DNA was removed using the TURBO DNA free kit (Thermo Fisher Scientific), in accordance with the manufacturer’s instructions. Reverse transcription was conducted using the High‐Capacity cDNA Reverse Transcription Kit (Thermo Fisher Scientific), followed by RT‐qPCR using the StepOnePlus™ Real‐Time PCR System (Thermo Fisher Scientific, Waltham, MA) with SYBR Green PCR master mix (Thermo Fisher Scientific). RT‐qPCR was conducted on BiP, PDI and bZIP60 as previously described (PMID 29441088) (Hamorsky *et al.*, 2015).

### Statistical analysis

All statistical analyses were conducted using GraphPad prism software. Differences in relative expression levels and differences in relative transcript abundance were both analyzed by one‐way ANOVA with Bonferroni’s multiple comparison tests.

## Conflicts of interest

E.M., A.M. and E.P.R. declare that they are named inventors on a patent application describing the co‐expression of chaperones to improve the production of heterologous proteins in plants (PA167643/PCT). G.P.L. declares that he is a named inventor on the granted patent WO 29087391 A1 which describes the pEAQ transient expression system used in this manuscript.

## Author contributions

EM, MV, JN, HP, DP, YM conducted the cloning and the expression work in plants. YJO conducted the RT‐qPCR analysis. EM led the experimental aspects of the project and compiled the manuscript. MvD contributed to experimental design. GPL supervised the expression work conducted at the John Innes Centre. NM supervised the experimental work conducted at the University of Louiseville School of Medicine. AM, RC, ER and ALW supervised the experimental work and contributed to experiment design. All authors provided feedback on the manuscript.

## Supporting information


**Figure S1 **Phenotype of *N. benthamiana* plants 3 days after infiltration with *A. tumefaciens* encoding human PDI.

**Figure S2 **Western blotting to detect expression of recombinant HIV gp140 following the co‐expression of (a) human BiP and (b) ERp57.

**Figure S3 **Co‐expression of human calnexin and calreticulin with influenza HA and dengue virus prME.
