# Supplementary material for: Co‐expression of human calreticulin significantly improves the production of HIV gp140 and other viral glycoproteins in plants
Source: Plant Biotechnol J. 2020 Mar 13;18(10):2109–17. doi: 10.1111/pbi.13369 (PMC7540014; doi:10.1111/pbi.13369)
Supplement: Supplementary file 1 — Figure S1 Phenotype of N. benthamiana plants 3 days after infiltration with A. tumefaciens encoding human PDI. [file PBI-18-2109-s001.pptx]

## Slide 1
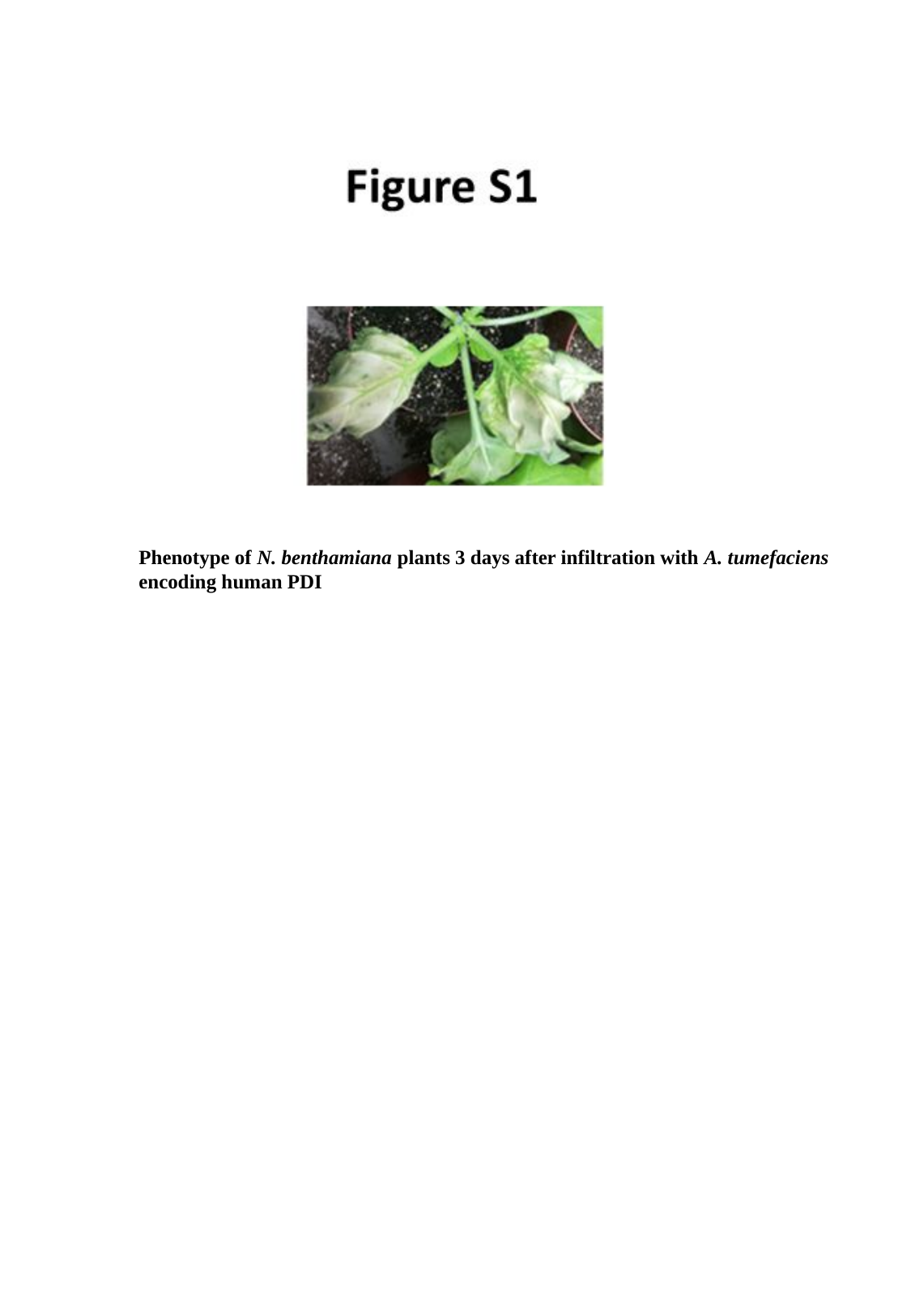

Phenotype of N. benthamiana plants 3 days after infiltration with A. tumefaciens encoding human PDI
