# Supplementary material for: Co‐expression of human calreticulin significantly improves the production of HIV gp140 and other viral glycoproteins in plants
Source: Plant Biotechnol J. 2020 Mar 13;18(10):2109–17. doi: 10.1111/pbi.13369 (PMC7540014; doi:10.1111/pbi.13369)
Supplement: Supplementary file 2 — Figure S2 Western blotting to detect expression of recombinant HIV gp140 following the co‐expression of (a) human BiP and (b) ERp57. [file PBI-18-2109-s002.pptx]

## Slide 1
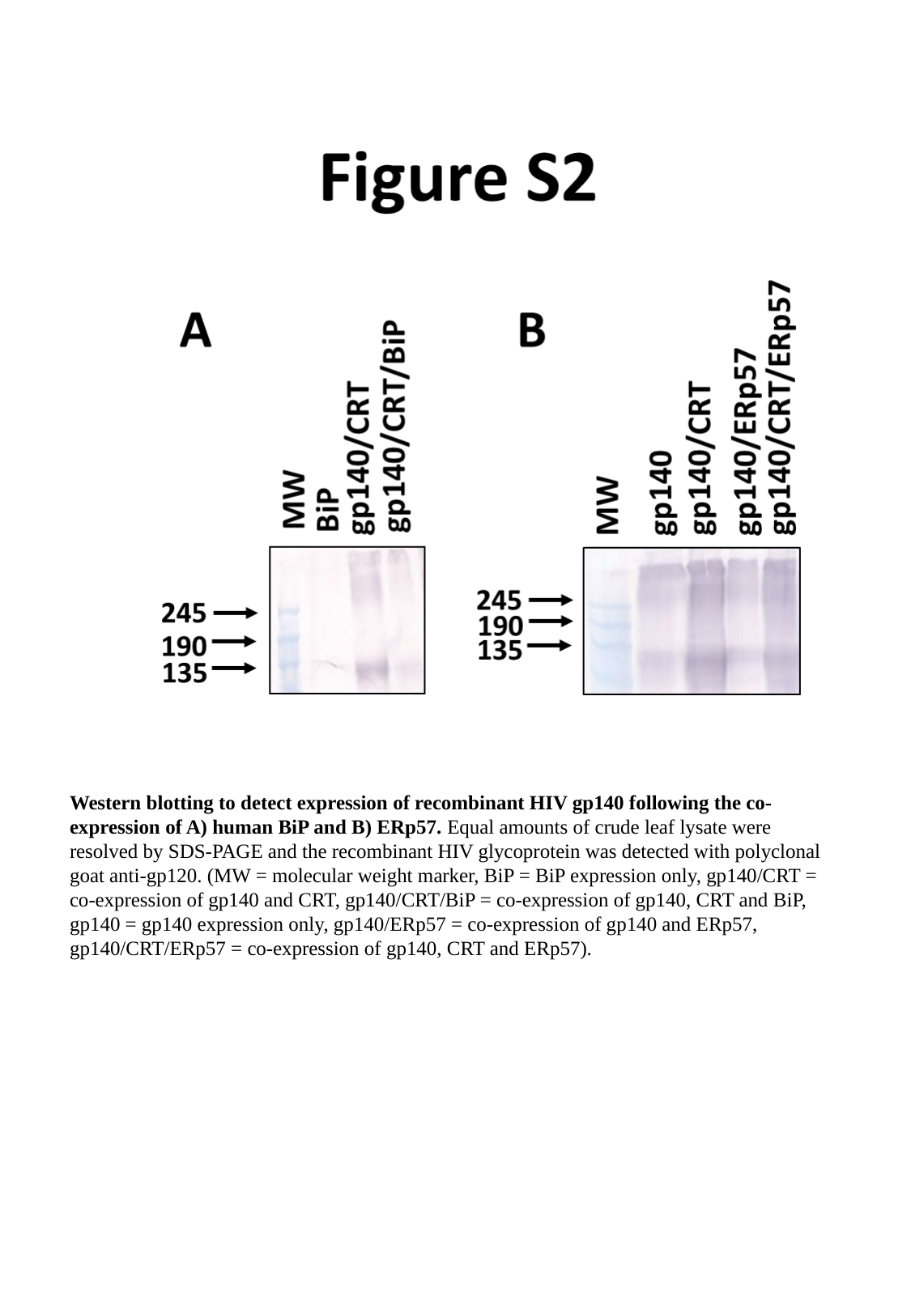

Western blotting to detect expression of recombinant HIV gp140 following the co-expression of A) human BiP and B) ERp57. Equal amounts of crude leaf lysate were resolved by SDS-PAGE and the recombinant HIV glycoprotein was detected with polyclonal goat anti-gp120. (MW = molecular weight marker, BiP = BiP expression only, gp140/CRT = co-expression of gp140 and CRT, gp140/CRT/BiP = co-expression of gp140, CRT and BiP, gp140 = gp140 expression only, gp140/ERp57 = co-expression of gp140 and ERp57, gp140/CRT/ERp57 = co-expression of gp140, CRT and ERp57).
