# Supplementary material for: Co‐expression of human calreticulin significantly improves the production of HIV gp140 and other viral glycoproteins in plants
Source: Plant Biotechnol J. 2020 Mar 13;18(10):2109–17. doi: 10.1111/pbi.13369 (PMC7540014; doi:10.1111/pbi.13369)
Supplement: Supplementary file 3 — Figure S3 Co‐expression of human calnexin and calreticulin with influenza HA and dengue virus prME. [file PBI-18-2109-s003.pptx]

## Slide 1
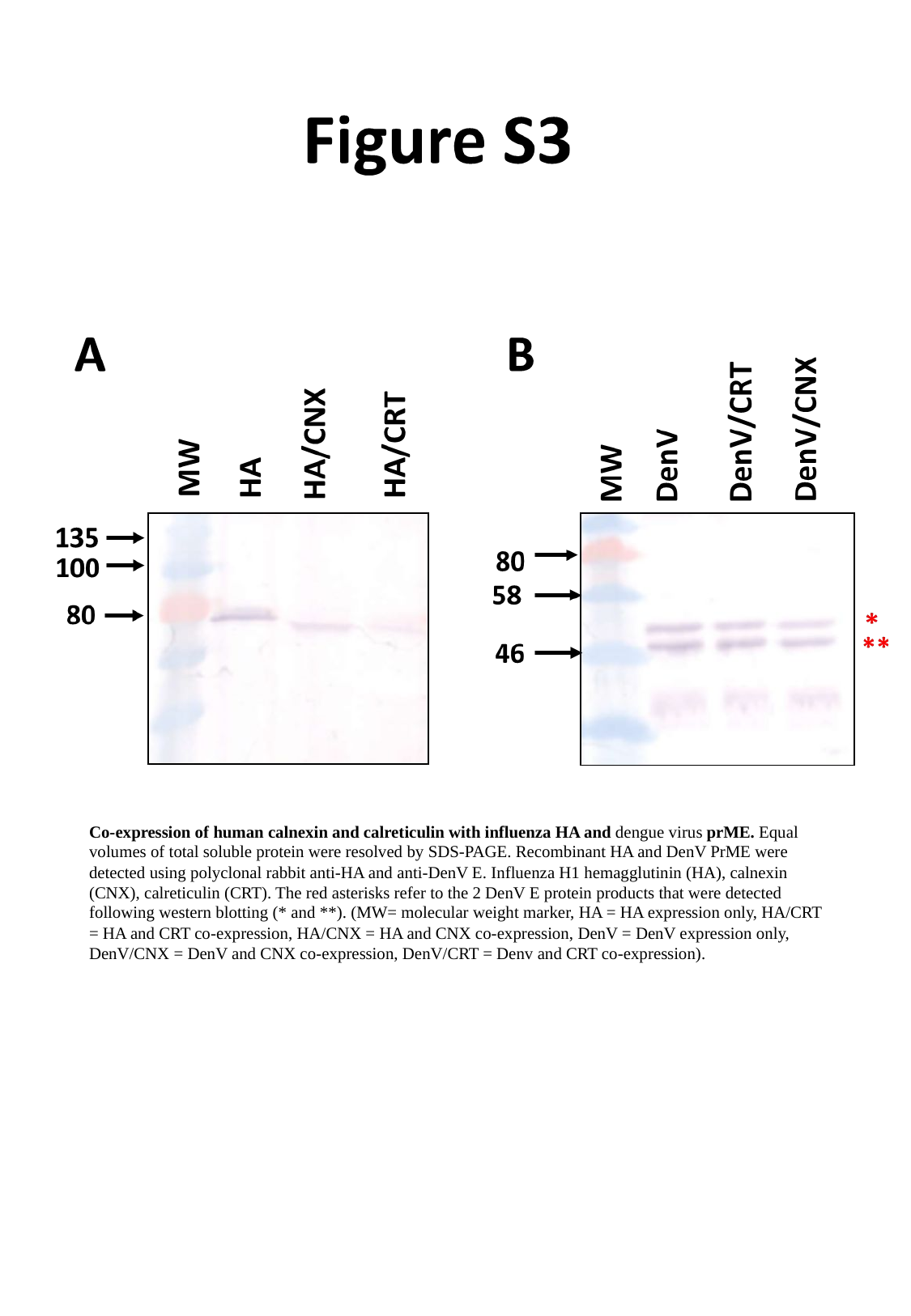

Co-expression of human calnexin and calreticulin with influenza HA and dengue virus prME. Equal volumes of total soluble protein were resolved by SDS-PAGE. Recombinant HA and DenV PrME were detected using polyclonal rabbit anti-HA and anti-DenV E. Influenza H1 hemagglutinin (HA), calnexin (CNX), calreticulin (CRT). The red asterisks refer to the 2 DenV E protein products that were detected following western blotting (* and **). (MW= molecular weight marker, HA = HA expression only, HA/CRT = HA and CRT co-expression, HA/CNX = HA and CNX co-expression, DenV = DenV expression only, DenV/CNX = DenV and CNX co-expression, DenV/CRT = Denv and CRT co-expression).
